# Circular Economy, Dairy Cow Feed Leftovers, and *Withania somnifera* Supplementation: Effects on Black Belly Ram’s Libido, Sperm Quality, Sexual Behavior, and Hemogram Values

**DOI:** 10.3390/biology13090656

**Published:** 2024-08-24

**Authors:** Andrés J. Rodriguez-Sánchez, Cesar A. Meza-Herrera, Angeles De Santiago-Miramontes, Cayetano Navarrete-Molina, Francisco G. Veliz-Deras, Julieta Z. Ordoñez-Morales, Jessica M. Flores-Salas, Ruben I. Marin-Tinoco

**Affiliations:** 1 Programa de Posgrado en Ciencias en Producción Agropecuaria, Universidad Autónoma Agraria Antonio Narro Unidad Laguna, Torreon 27054, Mexico; 2 Unidad Regional Universitaria de Zonas Áridas, Universidad Autónoma Chapingo, Mapimí 35230, Mexico; 3 Departmento de Química Area Tecnología Ambiental, Universidad Tecnológica de Rodeo, Rodeo 35760, Mexico; navarretemolina1977@gmail.com (C.N.-M.);; 4 Hospital Rural no. 162 Instituto Mexicano del Seguro Social, Rodeo 35760, Mexico

**Keywords:** hair sheep, dairy cows, feed surplus, ashwagandha supplementation, reproductive outcomes

## Abstract

**Simple Summary:**

In the Americas, the Comarca Lagunera (CL), located in North-arid Mexico, concentrates one of the main dairy cattle hubs (>500,000 Holstein cows) managed under highly technified-industrial schemes. Dairy cows’ nutrition is based on the use of total mixed rations; whereas feed rejection (i.e., 3–5%) has been observed, such feed-leftovers retain a high-pitched nutritional value. The CL also holds an emergent sheep industry, which exhibited the largest production value growth (i.e., >580%; 2010–2023). With that in mind and considering a circular economy perspective, we envisioned a research opportunity. Our working hypothesis states that a basal diet founded on such dairy cow feed-leftovers and aligned with short-term supplementation (i.e., 40 d) with *Withania somnifera* L. (Ashwagandha) will enhance some reproductive and behavioral outcomes in Black Belly rams. Such approach should bridge these two interesting animal production systems in the CL.

**Abstract:**

Considering a circular economy perspective, this study evaluates the possible effect of targeted short-term supplementation with *Withania somnifera* L. (WS; Ashwagandha) on ram’s seminal quality, socio-sexual behaviors, and blood constituents. Black Belly rams (n = 20) received a basal diet comprising feed-leftovers from dairy cows in the north-arid Mexico (i.e., Comarca Lagunera CL). The experimental units, with proven libido and fertility, were homogeneous in terms of age (3.41 ± 0.21 yr.), live weight (LW; 53.8 ± 3.3 kg), body condition (BC; 2.96 ± 0.01 units), initial sperm concentration (2387 ± 804 × 10^6^), and viability (23.9 ± 15.6%). Rams were randomly assigned during the transition reproductive period (i.e., May to Jun; 25° NL) to three treatment groups: non-supplemented control group (CONT; n = 6), low WS-supplemented (LWS; i.e., 100 mg kg LW^−1^ d^−1^ × 40 d; n = 7), and high-WS-supplemented (HWS; i.e., 200 mg kg LW^−1^ d^−1^ × 40 d; n = 7). The basal leftover diet was offered twice daily (0700 and 1600 h); the experimental period (EP) lasted 47 d. No differences (*p* > 0.05) among treatments occurred regarding LW and BCS at the onset of the EP. Whereas the greater scrotal circumference (SCRC, cm) arose in the LWS and CONT rams, an increased ejaculated volume (VOLEJA, mL) occurred in the WS-rams. A total of 5/9 (i.e., 55%) appetitive and 3/3 (i.e., 100%) consummatory sexual behaviors favored (*p* < 0.05) the WS-rams, particularly the HWS rams, towards the final EP. The same was true (*p* < 0.05) regarding the hemogram variables white blood cell count (×10^9^ cells L^−1^), hemoglobin concentration (g dL^−1^), and medium corpuscular volume (fL). This study, based on a rethink–reuse–reduce enquiry approach, enabled connectedness between two noteworthy animal systems in the CL: dairy cows and meat sheep schemes. Certainly, the use of dairy cow feed-leftovers aligned with the short-term supplementation with WS promoted enhanced testicular function, augmented seminal volume, and an increased sexual behavior in Black Belly rams in northern Mexico. Finally, while our research outcomes should enhance not only the resilience and sustainability of sheep production and the well-being of sheep-producers and their families, it may also embrace clinical translational applications.

## 1. Introduction

In the Americas, the Comarca Lagunera (CL)—an agroecological arid region located in northern Mexico, is one of the main dairy-cattle-producing regions. In 2023, with a total inventory of 500,000 Holstein cows and 245,000 milked cows, the CL generated 3000 million liters of milk, with a production value of around 1420 million USD [1,2]. Regarding small ruminants, between 1970–2023, whereas the national goat population showed a decrease of 7.5%, the sheep inventory increased 37%, with a final inventory of 8.8 million sheep. Five provinces (i.e., Mexico, Hidalgo, Veracruz, Jalisco, and Puebla) accounted for about 50% of the national sheep inventory, whereas the CL generated sheep meat production close to 180,000 kg, with a production value of 660,000 USD [1]. While the economic and productive importance of the dairy cow industry in the CL is beyond any doubt, it is worth it to state that the sheep productive and economic indicators in the CL become potentially important. During the period 2000–2023, the CL’s sheep inventory grew 318%, with concomitant increases in sheep-meat production (i.e., 600%) and the sheep-production economic value (i.e., 580%). Indeed, the sheep production displayed the largest census growth of the livestock sector in the CL [1].

Most of the dairy cattle clusters in the CL are managed under highly intensified industrial production schemes and fed with total mixed rations [3]. Whereas a feed rejection of 3–5% of the offered mixed diet has been reported, such surplus feed holds a high nutritional value. Previous research from our group has demonstrated a positive effect of the dairy cow feed-leftover use to enhance the out-of-season reproductive performance in goats under extensive conditions [4] and upon the generation of some goat out-of-season bioeconomic indices [5]. These studies suggest a kind of “*Robin Hood Effect*” [4] by dispersing some of the non-reusable energy–protein assets from a majorly unsustainable, wasteful linear bovine model [3] towards a goat extensive system, resembling a more circular, less linear model of production [6]. In addition, and from an economic and biologic standpoint, reproductive efficiency is essential to increase the pregnancy rate and consequently augment the viability and resilience of any animal system, as well as the economic return to the producer. Certainly, the male reproductive efficiency is central to achieve greater viability not only from a productive, but also from a genetic and economic return, standpoint. On this regard, and under a clean, green, and ethical intervention approach, the use of various phytoextracts to improve not only the level of production, but also the male reproductive performance and sexual behavior in diverse animal species, has grown [7,8,9,10]. Moreover, low reproductive performance in males can be triggered by diverse hormonal, nutritional, and infectious imbalances, among others [11].

The use of nutraceuticals to increase libido and sperm quality has significantly emerged during the last decades [12,13]. As reported, the nutritional content of different plants plays an essential role due to their medicinal, nutritional, and therapeutic properties [10]. From a therapeutic standpoint, consumption of the roots of a small evergreen shrub called *Withania somnifera* [WS; Ashwagandha] has shown positive effects in animal production [12]. This WS-root has demonstrated anti-inflammatory, antitumor, anti-stress, antioxidant, and immunomodulator effects, improving hematopoiesis, while it seems to act on the endocrine, cardiopulmonary, and central nervous systems [12,14,15,16,17]. In addition, the sperm quality of infertile males can be improved by the oral intake of WS-root extracts. Certainly, the extracts of the WS-root inhibit lipid peroxidation, improve sperm counts and motility, and regulate reproductive hormone levels, with collateral benefits on the health status [8,15]. The use of such adaptogens also dynamizes the economy in diverse animal production systems [8,18].

Sheep farming, as an emergent, yet with high expansion potential, animal industry in the CL, requires the development of initiatives to increase reproductive rates to significantly impact the sheep´s productive performance. In addition, there is a large opportunity to use the rejected mixed ration from the highly industrialized dairy cow production system in the CL. Therefore, we visualize an interesting research opportunity based on a rethink–reuse–reduce enquiry approach, looking for connectedness between these two interesting ruminant production schemes. Hence, we hypothesized that the use of feed-leftovers from the dairy cow industry as a basal diet aligned with the short-term supplementation with *W. somnifera* (i.e., WS) will promote enhancement of the reproductive, behavioral, and hemogram values in male sheep. To test this hypothesis, our aim was to evaluate the possible effect of this basal diet + WS-supplementation on the testicular size and function, sperm quality, sexual behavior (i.e., appetitive and consummatory), and hemogram values in Black Belly rams in northern Mexico.

## 2. Materials and Methods

### 2.1. General

All methods, experimental procedures, and management conducted in this study adhered strictly to guidelines for the ethical treatment, care, and welfare of animals in research at both the international [19] and national [20] levels. In this study, all the reported procedures aligned with conventional veterinary practices; blood and semen sampling were performed by trained veterinarians. In addition, the study received institutional endorsement under the reference number UAAAN-UL-425502002-2743.

### 2.2. Location, Environmental Conditions, Experimental Groups, and Management

**Figure 1:**
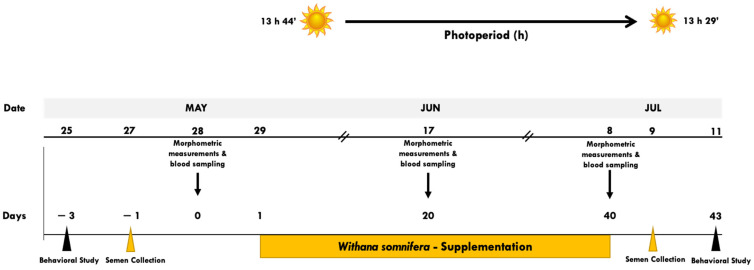
Schematic representation of the experimental protocol. Black Belly rams (n = 20), with proven libido and fertility, homogeneous in terms of age (3.41 ± 0.21 years), live weight (LW; 53.8 ± 3.3 kg), body condition (BC; 2.96 ± 0.01 units), and initial sperm concentration (2387 ± 804 × 10^6^) and viability (23.9 ± 15.6%) were fed twice daily (0700 and 1600 h), with a basal diet comprising the feed-leftovers from an industrial dairy cow enterprise. Rams were randomly assigned during the transition reproductive period (i.e., May–Jun–Jul; 25° NL) to three treatment groups: non-supplemented control group (CONT, n = 6), low WS-supplemented (LWS; i.e., 100 mg kg LW^−1^ d^−1^; n = 7), and high-supplemented (HWS; i.e., 200 mg kg LW^−1^ d^−1^; n = 7). While the experimental period (EP) lasted 47 d, the response variables included corporal and testicular measurements, seminal evaluations, socio-sexual behavior (i.e., appetitive and consummatory), and hemogram quantifications. On May 28, Jun 17, and Jul 08; (d-1, 20 and 40 of the EP), LW, BC, scrotal circumference, and odor intensity were registered; blood samples were also collected to build the hemogram response. On days-3 and 43 of the EP, behavioral male-to-female tests were carried out (0800 h, during 15 min; each day) quantifying the number of sexual (i.e., appetitive and consummatory) and non-sexual behaviors. Also, on day-1 and 41, semen samples were collected to quantify seminal quantity and quality. No indication of any health-related issues was observed along with the EP.

The study was performed on a sheep herd (i.e., El Milagro) managed under intensive conditions, located in Matamoros, Coahuila, belonging to the CL in northern Mexico (25° NL, 103° WL, 1110 m altitude). The region is defined as a semi-arid ecotype, with an average annual temperature of 23.8 °C, a maximum of 41 °C in Jun, and a minimum of −1 °C in Dec and Jan. The photoperiod fluctuates from 13 h 41 min in the summer solstice down to 10 h 19 min at the winter solstice, having an average annual rainfall of 230 mm [21]. The prevailing photoperiod and the chronology of the main activities carried out, along with the experimental period, are shown in Figure 1.

Adult male sheep of the Black Belly breed (n = 20), with proven libido and fertility, homogeneous in terms of age (3.41 ± 0.21 year, live weight (LW; 53.8 ± 3.3 kg), body condition (BC; 2.96 ± 0.01 units), and initial concentration (2387 ± 804 × 10^6^) and sperm viability (23.9 ± 15.6%) were randomly assigned to three experimental groups: non-supplemented control group (CONT; n = 6), low-supplemented group (LWS; i.e., 100 mg kg LW^−1^ d^−1^ × 40 d; n = 7), and high supplemented group (HWS; i.e., 200 mg kg LW^−1^ d^−1^ × 40 d; n = 7). The experimental groups received a basal diet twice daily (0700 and 1600 h) comprising the feed-leftovers from an industrial, highly technified dairy cow enterprise and composed of a mixture of alfalfa hay, corn silage, rolled corn grain, soybean meal, cottonseed meal, and a vitamin–mineral premix. Both the physical and chemical conformation of the basal diet (i.e., dairy cow feed-leftovers), as well as the management of the offered basal diet, were previously defined [4,5]. Briefly, the feed-leftovers from a dairy cow enterprise were collected daily (i.e., 0700 h) and brought to the ram´s corrals to be individually offered to the WS-supplemented rams. Rams were dewormed one month prior to the experimental period and received fat-soluble vitamins (A 500,000 UI; D 75,000 UI; E: 50 UI). In addition, cleaning, attempting to control the fly population, was carried in both the general corrals and the experimental pens; clean fresh water was provided ad libitum. Rams had a three-week adaptation period to the basal diet, prior to the onset of the EP. The chemical and physical composition of the basal diet is shown in Table 1.

### 2.3. Response Variables: Corporal, Odor, Testicular, and Seminal Evaluations

During the transition reproductive period (i.e., May–Jun) the response variables evaluated were recorded on days 0 (i.e., initial) and 40 (i.e., final) of the EP, and the following were considered: live weight (LW, kg), body condition (BC, units), scrotal circumference (SC, cm), odor intensity (ODOR, units); blood samples were collected to quantify hemogram values. Whereas the LW was registered by using a digital scale with a capacity of 150 kg and a minimum of 50 g (WH-C 100, Guangdong, China), BC was evaluated via palpation of the thickness of the muscle and adipose mass. The BC-evaluated areas considered the space between the spinous and transverse processes of the lumbar vertebrae, with a range of 1 to 4 (1 = emaciated and 4 = obese) according to the technique previously described [24]. Also, with respect to the libido and semen quality variables, the following issues were registered: latency to ejaculation (LATEJA, seconds), volume ejaculated (VOLEJA, mL), sperm concertation (SPERCON, ×10^6^), mass motility (MASMOT, units), mass progressive motility (MASPROG, %), and sperm viability (VIABILI, %). These variables were recorded on days-1 and 40 of the EP. Whereas the SC was registered by measuring the widest point of the testes with a flexible tape measure graduated in millimeters [25], odor intensity was evaluated using a previously described technique [24]. Briefly, the back of the base of the horns was sniffed at 15 cm (range 0–3; 0, neutral odor or equal to females or neutered male; 1, light male odor; 2, moderate male taint; and 3, intense male taint).

To evaluate semen quality, it was collected from each male with the help of an artificial vagina using an estrogenized-stimulated female (i.e., 2 mg of estradiol cypionate via IM, every third day; Zoetis^®^, Mexico City, Mexico). Previously, the water in the artificial vagina was preheated (i.e., 45 °C). Then, the semen was collected in graduated glass tubes, which were immediately immersed in a water bath at 37 °C for subsequent analyses over the next 10 min. The latency to ejaculate (i.e., seconds) was defined as an indirect quantification of the ram libido and considered the period from the time the male was exposed to the female in estrus to the time the ram ejaculated into the artificial vagina. Each male was exposed to the female for 300 s; after this time, if the male did not mount the female, it was considered rejection to ejaculate. Next, macro and microscopic assessments were carried out. The ejaculated volume (mL) was quantified directly in the conical collection tube, graduated with optically visible intervals of 0.1 mL. Semen color was recorded parallel to the quantification of volume directly, using the glass tube for semen collection. The seminal color collected was classified as white, milky-white, or creamy white.

The sperm concentration (×10^6^ mL^−1^) was determined via photometric analysis (Spermacue^®^, 12300/0500 Minitub, Landshut, Germany), according to Olivera-Muzante and collaborators in 2011 [26]), using undiluted semen, expressing itself as ×10^6^ cells mL^−1^. The total number of ejaculated spermatozoa (units) was calculated considering the concentration of spermatozoa per mL, multiplied by the total volume ejaculated, and expressed as ×10^6^ cells. Mass motility (%) was evaluated considering a scale of 1 to 5, where 1 = 20% and 5 = 100% motile spermatozoa, as previously suggested [11]. Briefly, a preheated slide (37 °C) was used, and then, 10 μL of undiluted semen was added, and mass motility was quantified using a phase contrast a microscope (Olympis CX43, Minitube, trinocular and heated stage, Tiefenbach, Germany). Progressive motility was determined based on the proportion of progressively motile spermatozoa. Briefly, 10 μL of undiluted semen was placed on a preheated slide, and then, a coverslip slide was placed and observed under a microscope (Olympis CX43, Minitube, trinocular, with a 40× objective, Tiefenbach, Germany). Finally, sperm viability was evaluated by means of the eosin–nigrosin staining technique [9]. Briefly, 200 cells per sample were observed using an optical microscope, using a 100× objective, calculating the percentage of live cells (undyed) and dead cells (stained pink). All evaluations were carried out by the same technician.

### 2.4. Appetitive-Consummatory Sexual and Non-Sexual Behavioral Evaluations

The response variables to evaluate sexual behavior were recorded on days 0 (i.e., initial) and 40 (i.e., final) of the experimental period. Sexual behavioral tests were performed on each male on two consecutive days. Each ram was exposed to one estrogenized female during 15 min per test; the numbers of previously proposed sexual behaviors were quantified [27,28,29]. Appetitive sexual behavior considered approaches (APRO, n), kicking (KICK, n), ano-genital sniffing (ANGESNI, n), corporal sniffing (CORPSNI, n), flehmen (FLE, n), flehmen + sniffing (FLESNI, n), flehmen + urine sniffing (FLEURSNI, n), pennis extrusion (PENEXTR, n), and attempt mounting (ATTMO, n). Regarding consummatory sexual behavior, the following response variables were evaluated: mount + pennis extrusion (MOPENEXTR, n), mount + pennis intromission (MOPENINTRO, n), and mount + ejaculation (MOEJACU, n). Finally, other non-sexual behaviors were also registered: [low vocalization (LVOCA, n), high vocalization (HVOCA, n), standing position (STAND, n), as well as isolation + attempt to scape (ISOATTESC, n).

### 2.5. Blood Sampling: Quantification of the Ram Blood Count Values—Hemogram

Along with the experimental period, all rams were blood-sampled to determine hematic count values. Blood samples (i.e., 5 mL) were collected via jugular venipuncture with 9 mL BD Vacutainer^®^ tubes (Broken Bow, NE, USA). Tubes contained 7.2 mg of spry-dried K2EDTA on its inner wall, which acts as an anticoagulant, binding the calcium ions and interrupting the blood clotting process to assess the complete blood-plasma count. Blood samples for hemogram were collected at 0800 h under fasting conditions. Then, plasma samples were used to quantify the complete blood count with an automated hematology analyzer (Hemalyzer 1000; Minneapolis, MN, USA), performed by a trained technician. The blood response variables included the white blood cell count (WBCC, ×10^9^ cells L^−1^), red blood cell count (RBCC, ×10^12^ cells L^−1^), hemoglobin concentration (HbC, g dL^−1^), hematocrit (Ht, %), medium corpuscular volume (MCV, fL), mean corpuscular hemoglobin (MCHb, pg), mean corpuscular hemoglobin concentration (MCHbC, g dL^−1^), and red blood cell distribution width (RBCDW, %).

### 2.6. Statistical Analyses

The corporal, testicular, seminal, sexual-behavioral, and hemogram response variables were analyzed by using linear mixed models for repeated measurements in the same animal across time, by means of the PROC MIXED procedure. In the final model, the fixed effect was the supplementation level, whereas the repeated measurement was the sampling period; each period represented the measurement of diverse response variables of the same experimental unit under a different time condition. The random effect was the ram´s ID within the supplementation level; each ram was considered an experimental unit. Because discontinuous or categorical variables did not fit the normal distribution, they were analyzed by means of the GENMOD procedure, using the LOGIT function to reduce skewness. When significant F values were observed among the response variables, mean separation among experimental groups considered the LSMEAN/PDIFF procedure of SAS; a statistical difference was considered at a value of *p* ≤ 0.05. All statistical analyses were solved using the SAS statistical package (SAS Inst. Inc. Ver. 9.4, 2016, Cary, NC, USA). The reported results in this study are expressed in lsmeans ± sem.

## 3. Results

### 3.1. Withania somnifera Supplementation and Corporal, Testicular, and Semen Responses

Table 2 shows the corporal, testicular, and seminal variables. Differences (*p* < 0.05) among treatments occurred with respect to the response variables: (1) SCRC, with the lowest value in the HWS group, (2) ODOR, favoring to the HWS group, and (3) VOLEJA, with the largest volume occurring in the LWS group. In contrast, no differences (*p* > 0.05) among treatments were observed regarding the following variables: LW (55.4 ± 3.16 kg), BCS (3.0 ± 0.06 units), LATEJA (134.5 ± 45.16 s), SPERCON (2582.3 ± 708.3 × 10^6^), MASMOT (1.93 ± 0.7 units), MASPROG (38.3 ± 14.2%), and VIABIL (33.3 ± 13.1%).

### 3.2. Withania somnifera Supplementation and Sexual and Non-Sexual Behavioral Responses

Appetitive and consummatory sexual behaviors, as well as other social, non-sexual behaviors are shown in Table 3. From an appetitive behavior perspective, the ANGESNI variable differed (*p* < 0.05) among groups, favoring the HWS. In contrast, the variables APRO (6.9 ± 2.26 n), KICK (3.6 ± 1.43 n), CORPSNI (5.4 ± 2.3 n), FLE (0.56 ± 0.5 n), FLESNI (1.4 ± 0.8 n), FLEURSNI (0.76 ± 0.5 n), PENEXTR (2.56 ± 1.3 n), and ATTMO (2.23 ± 1.2 n) did not differ (*p* > 0.05) among the experimental groups. Regarding the consummatory sexual behaviors, while MOPENEXTR favored (*p* < 0.05) the HWS group, no differences (*p* > 0.05) occurred for MOPENINTRO (1.5 ± 0.50 n) and MOEJACU (0.76 ± 0.23 n) among treatments. When considering the quantification of other non-sexual behaviors, such as low vocalizations (LVOCA, n), high vocalizations (HVOCA, n), standing position (STAND, n), and isolation + attempt to escape (ISOATTESC, n), none of them accounted for differences (*p* > 0.05) among experimental groups.

### 3.3. Withania somnifera Supplementation and Some Hemogram Responses

The least-square means ± standard error for the hemogram response variables are shown in Table 4; HbC (g dL^−1^) was the only hemogram variable that differed (*p* < 0.05) among treatments, favoring those rams supplemented with *W. somnifera* either at low (i.e., 100 mg d^−1^) or high (i.e., 200 mg d^−1^) levels, as compared with the non-supplemented Black Belly rams (i.e., CONT). In contrast, the hemogram response variables WBCC (118.6 ± 4.2 × 10^9^ cells L^−1^), RBCC, 3.06 ± 0.1 × 10^12^ cells L^−1^), Ht (12.2 ± 0.7%), MCV (39.7 ± 0.1 fL), MCHb (48.1 ± 2.7 pg), MCHbC (121.5 ± 6.9 g dL^−1^), and RBCDW (14.4 ± 0.2%) did not differ (*p* > 0.05) among Black Belly rams irrespectively of the WS-supplementation level.

### 3.4. Supplementation by Time Interaction—Corporal, Testicular, and Seminal Responses

The least-square means ± standard error, as affected by the simple effect of the supplementation by time interaction regarding corporal, testicular, and seminal variables, are presented in Table 5. Since only four response variables from this dataset (i.e., BCS, units; SCRC, cm; ODOR, u; and VOLEJA, mL) were affected (*p* < 0.05) by the treatment-by-time interaction, other blood variables were not included. Certainly, such non-included response variables were only affected by the main effect of the supplementation level as seen in Table 2. Regarding the response variables affected by the simple effect of the treatment-by-time interaction, differences (*p* < 0.05) emerged among treatments regarding the following: (a) BCS, observing the largest value (*p* < 0.01) towards the final part of the experimental period in the HWS-Black Belly rams; (b) SCRC, with the larger values occurring in the LWS and CONT groups; (c) ODOR, favoring the HWS-Black Belly rams; and (d) VOLEJA, with the larger volumes occurring in the *W. somnifera*-supplemented rams. Interestingly, despite the HWS-rams showing the lowest SCRC, they showed an increased VOLEJA (*p* < 0.01) regarding the CONT, which depicted increased SCRC values regarding the HWS-Black Belly rams.

### 3.5. Supplementation by Time Interaction—Sexual and Non-Sexual Behavioral Responses

Information regarding the effect of the supplementation level by time interaction affecting (*p* < 0.05) the performance of both appetitive and consummatory sexual behaviors, as well as other non-sexual behaviors, throughout the experimental period (i.e., simple effect of the treatment interaction by time) is presented in Table 6.

Interestingly, as seen in Table 6, a total of 5/9 (i.e., 55%) appetitive sexual behaviors and 3/3 (i.e., 100%) consummatory sexual behaviors favored (*p* < 0.05) the Black Belly rams supplemented with *W. somnifera*, particularly in the HWS group, towards the end of the experimental period. On the other hand, the FLE response variable showed a trend (*p* = 0.07) like its appetitive counterparts with the highest values not only towards the end of the study but also in those groups supplemented with *W. somnifera*. Finally, non-sexual behaviors, low-range vocalizations (*p* < 0.01), and aggressions + escape attempts (*p* < 0.05) were also affected by the effect of the interaction supplementation by time, favoring the HWS group.

### 3.6. Supplementation by Time Interaction—Some Hemogram Responses

According to the variables presented in Table 7, the highest (*p* < 0.05) WBCC values occurred in the HWS-Black Belly rams. A similar trend was followed by the HbC and MCV variables; the highest values were observed in the EP´s intermediate and final phases in those males receiving short-term supplementation with *W. somnifera* (*p* < 0.05). The other blood variables are not included in this table, because they were only affected by the main effect of the supplementation level (Table 4).

## 4. Discussion

Our working hypothesis stated that short-term *W. somnifera* supplementation (i.e., 40 d) generates an enhancement not only of the testicular size and function, but also an augmented sperm quality and quantity, with an increased odor intensity and socio-sexual behaviors (i.e., appetitive and consummatory) in Black Belly rams fed with feed-leftovers from a dairy cow industry as a basal diet in northern-arid Mexico. According to the main outcomes from this study, this working hypothesis is not rejected. In sires, either rams, bucks, or stallions, semen quality, libido, and the hemogram are important variables to define male reproductive fitness. Whereas sperm and blood cells appear to be among those cellular lines pointedly susceptible to metabolic and environmental influences, the interaction among diet, micronutrients, endogenous biochemical pathways, and reproductive-seminal outcomes in ruminants has been established [30]. In our study, an antioxidant role of WS may have played a supportive role, considering the high metabolic activity depicted by both sperm and blood cells, resulting in an increased cell turn over and therefore a large susceptibility to oxidative stress. Indeed, these two cell lines are expressly prone to subcellular changes generated by an augmented metabolic rate and, in turn, the release of reactive oxygen species (ROS) [31,32]. Therefore, WS-supplementation may have acted as a ROS-scavenger, enhancing the seminal and hemogram cellular outcomes. The last is in a pathway still to be defined but certainly enhanced by increases in serum testosterone concentrations. Such scenario may have influenced both appetitive and consummatory sexual behaviors in those Back Belly rams supplemented with WS, during the sexual resting–transition season (i.e., May-Jun-Jul; 25° N) in the CL.

### 4.1. Withania somnifera Supplementation and Some Corporal, Testicular, and Seminal Responses

While no differences among treatments occurred regarding LW and BCS at the onset of the EP, differences emerged by the end of the EP (i.e., BCS and ODOR); the largest values occurred in the HWS rams. The observed outcomes of this study, in particular the positive effect of WS-supplementation on SCRC and VOLEJA, certainly acquire special interest. As reported, WS not only induces testicular development [14] and augmented testosterone synthesis [Δ = 72 ng dL^−1^ (WS) vs. Δ = 5.4 ng dL^−1^ (CONT)] [16], but also enhanced semen quality by managing the optimum levels of essential amino acids, citrate, and lactate in seminal plasma [14]. Both reactive oxygen and nitrogen species are extremely responsive free radicals generated at subcellular testicular compartments, mainly mitochondria. Thus, the excessive production of free radicals promotes tissue damage and cell death because of a reduction in the antioxidant status [33,34]. Moreover, lipid peroxidation exerts damage to the sperm cellular membrane, engendering a significant reduction in fertility potentials. In addition, oxidative stress also impairs sperm viability, since under such scenarios, the unsaturated fatty acid content is naturally augmented in the cytoplasm [34]. Furthermore, ROS cause male infertility by preventing spermatogenesis. Even more, ROS compromise the sperm architecture and function, as well as sperm motility, viability, acrosome reaction, and sperm–ovum interactions. In turn, ROS reduce fertilization while diminishing implantation rates [35]. Therefore, a reduction in the excessive ROS levels is, certainly, a way to overcome or diminish such reproductive harms.

Further, and in a very interesting fashion, despite the HWS-rams exhibiting a diminished SCRC (i.e., a 13% reduction) regarding both CONT and LWS groups, the HWS-rams doubled the ejaculated volume as compared to the CONT-rams, with no differences among groups concerning the sperm concentration (i.e., 2582 sperms ×10^6^ mL^−1^). Therefore, an interesting question emerges from our study. Is it possible that the augmented SCRC and VOLTEJA values observed in the WS-rams can be extended to an in vivo (i.e., either direct mount or fresh semen) or in vitro (i.e., frozen semen) fertilizing potential? The last is central since the preparation of semen for assisted reproductive techniques requires the elimination of seminal plasma, which has a high amount of antioxidant molecules and enzymes. As the semen lacks its own protection against free radicals, they have a high risk of damage generated by oxidative stress [7,13]. Moreover, sperm cryopreservation allows for sperm storage. However, this process is also associated with the production of oxygen free radicals, which subsequently leads to lipid peroxidation and decreased sperm parameters [36]. Even when the level of ROS is pathologically augmented, antioxidant compounds start to work, aiding to lessen oxidative detriment, healing, or even prevent it [37]. Therefore, the assessment of the possible extended positive action of the WS supplementation upon the in vivo and in vitro seminal fertilizing potential emerges as an interesting research question that warrants further studies.

In an elegant study comparing the use of different phytoextracts in male rats, with WS among these, no differences (*p* > 0.05) regarding body weight, as well as absolute (g) and relative (%) reproductive organ weights (i.e., testis, whole epididymis, vas deferens, seminal vesicles, and ventral prostate) occurred between the WS and CONT groups. The same was true (*p* > 0.05) regarding total sperm motility (%) and abnormality rate (%; head, tail, and total). Nonetheless, the WS-supplemented males shown an increased (*p* < 0.01) sperm concentration regarding the CONT-rams (i.e., 154.8 vs. 110.3 × 10^6^ Right Cauda^−1^) [38]. Our research outcomes arise as the importance about the use of WS as an antioxidant adjuvant, to prevent the onset of disturbances in case of augmented metabolic activity, such as that observed previously (i.e., transition period) and during the breeding season. In rams, the spermatogenesis process (i.e., spermatocytogenesis, meiosis, and spermiogenesis) lasts around 60 days. Therefore, it could be tempting to propose that the duration of the WS-supplementation period (i.e., 40 d) was not sufficient to promote more substantial effects on the seminal outcomes from a quality and quantity perspective. Nonetheless, if we also consider the asynchronous nature of the spermatogenesis cycle, a restricted period effect of the WS-supplementation may be pondered as having a low impact. Undoubtedly, a 60 d WS-supplementation period would have defined a better and stronger response regarding the quality–quantity of semen in the Black Belly rams. Certainly, supplementation of at least 60 days, parallel to the positive effect of the 45 d WS-supplementation observed on the ejaculated volume, would possibly have been extended to other response variables, in particular, those defining seminal quality and even the libido level (i.e., LATEJA (s), SPERCON (×10^6^ mL^−1^), MASMOT (units), MASPROG (%), and VIABILI (%). Therefore, further studies must consider this situation.

### 4.2. Withania somnifera Supplementation and Some Sexual and Non-Sexual Behavioral Responses

Sexual behavior is a complex centralized controlled process that involves a myriad of neurotransmitters and neuropeptides. While the neural circuitry mediating sexual motivation and behavior overlaps in both sexes [39], the specific mating actions are sexually dimorphic yet strongly gonadal–endocrine-dependent in both sexes [40,41]. Certainly, gonadal hormones have an essential role in brain sexual differentiation at the fetal and perinatal stages by shaping sexually dimorphic neural circuits [39,40,41]. Later, gonadal hormones activate such sexual circuitry, promoting the expression of the relevant sex-typical behavior [39,40,41,42].

Withanolids are steroidal lactones (i.e., withaferin A and withanolide D) and are the main bioactive compounds present in WS; they target diverse biomolecules and are responsible for diverse pharmacological actions [17,43]. Testosterone is one of the main products of the steroidogenic pathway; its optimal level improves spermatogenesis, libido, energy, bone density, erectile function, and muscle strength [41,42,44]. In humans, the effect of WS supplementation (i.e., 8 weeks) was evaluated based on the androgen profile and various sexual domains. The response variables were registered at the initial and final week of the supplementation period and included serum testosterone, sexual fantasies, sexual arousal, sexual behavior, libido, ejaculation, and the total sexual score. While all these variables favored (*p* < 0.05) the WS-males, a substantial increase (*p* < 0.05) during the final assessment (i.e., week 8th) occurred in all the evaluated variables [16]. In another study comparing the use of different phytoextracts in male rats, with WS among these, all the sexual behavioral response variables [i.e., mounting latency (sec) and frequency (n), as well as intromission latency (sec) and frequency (n)] favored (*p* < 0.01) the WS-supplemented males [38]. Intriguingly, however, while no differences (*p* > 0.05) for FSH and LH occurred between the WS and CONT groups, the WS-males displayed the largest (*p* < 0.01) testosterone concentration (i.e., 3.5 vs. 2.2 ± 0.1 ng mL^−1^). Moreover, when quantifying the concentration of malonaldehyde (MDA), an index of lipid peroxidation and oxidative stress, the CONT-males disclosed the largest values, either in serum (i.e., 0.54 vs. 0.44 ± 0.05 µmol L^−1^) or in the testis (i.e., 1.64 vs. 1.51 ± 0.09 nmol g^−1^) regarding the WS-supplemented males [38]. The last confirms the central role of WS as an adaptogen that reduces lipid peroxidation and oxidative stress.

Sexual behaviors are often conceptualized in two classes: appetitive and consummatory [28,29,45]. In our study, a total of 10 out of 16 (i.e., 62.5%) socio-sexual behaviors were linked to the short-term WS-supplementation. From these behaviors, 5/9 (i.e., 55%) were defined as appetitive, but 3/3 (i.e., 100%) were defined as consummatory sexual behaviors; all of these favored (*p* < 0.05) the WS-rams, particularly the HWS rams around the end of the EP. WS-influenced socio-sexual behaviors included appetitive behaviors, such as approaches, kicking, ano-genital sniffing, corporal sniffing, and flehmen, consummatory behaviors, like mount + pennis extrusion, mount + pennis intromission, and mount + ejaculation, and non-sexual behaviors, such as low vocalizations and isolation + attempt to escape.

### 4.3. Withania somnifera Supplementation and Some Hemogram Responses

Blood is constituted of formed elements, such as red blood cells, white blood cells, and platelets, which are suspended in a fluid medium termed plasma. Plasma is obtained via the centrifugation of blood that is prevented from clotting and comprises 55–66% of the total volume. In a very interesting study comparing the use of different phytoextracts in male rats, with WS among these, based on some hemogram response variables, no differences (*p* > 0.05) arose among WS-males, the control positive sildenafil, and the control negative standard group [38]. In our study, the hemogram response variables white blood cell count (WBCC, ×10^9^ cells L^−1^), hemoglobin concentration (HbC, g dL^−1^), and medium corpuscular volume (MCV, fL) were positively affected by the short-term WS-supplementation, although all of them were within the normal range values for sheep. While an increased WBCC may denote a normal response to reduce inflammation processes, an augmented HbC may suggest a natural response to an amplified metabolic rate, whereas MCV is an indicator of the RBC average size; augmented values could be related to low iron levels.

Prior to close the Discussion of our study, and contrasting our results with previous studies cited in the scientific literature, we still have two pending assignments. The first one is to propose some possible mechanisms of action through which the WS short-term supplementation could have affected most of the response variables in our study. While we certainly have a fragmentary knowledge of such respects, a starting point is to gain a broader insight regarding the molecular mechanisms involved in the antioxidant response exerted by WS in the male reproductive tissues. For this respect, the Nrf2 signaling pathway (i.e., erythroid 2-related factor 2) has been proposed as a transcription factor that promotes the expression of different and crucial antioxidant genes [46,47]. In addition, NF-kB, an enhancer binding-transcription factor aligned to the immune, cell proliferation, and apoptosis responses has shown not only important pro-survival actions through the induced transcription of several antiapoptotic genes [48], but it has been related to the downregulation of neuroinflammatory genes [49].

Therefore, the possible WS-supplementation effect on the expression of NFkB and Nrf2 protein levels, along with some components of the male reproductive tract, should be an important point of departure. Accordingly, WS supplementation promoted the augmented protein expression of Nrf2 in the vas deferens, epididymis, testes, and prostate, without major effects regarding the protein expression of NFkB. Another plausible WS-mechanism of the action could be its role as a facilitator, either in vitro and in vivo, based on the expression and release of GnRH throughout GABAergic neurons [50]. In addition, the action of WS supplementation may also act through withanolides, probably derived from cholesterol; withanolides involve a group of C28-steroidal lactone triterpenoids that resemble the structure of testosterone [51]. Therefore, considering such previous research outcomes merged with our research results, we can observe a feasible and interesting way to better understand how the WS-supplementation increased spermatogenesis, odor intensity, and libido, with all of them linked to an enlarged sexual behavior, either appetitive or consummatory, observed in the ashwagandha-supplemented Black Belly rams. Unquestionably, these outlined possible mechanisms of WS action on male reproductive and sexual outcomes, although tempting and persuasive, remain to be scientifically proven.

The second pending assignment relates to a comment on the potential impact that bridging of the dairy cow production system and the meat sheep production system may have on the animal industry in the CL from a socio-economic standpoint. In this regard, we start from the volume of leftovers available from the dairy cattle industry, only considering the rejection (i.e., 4–5%) of the cows in production (i.e., only milking cows, excluding dry cows). These values range from 157,389 to 209,236 tons on a wet basis, equivalent to 80,654 to 100,818 tons on a dry basis. If we consider a conservative average sheep LW of 50 kg (i.e., males and females) with an average consumption of 3% LW, the last is equivalent to an annualized consumption of 1.14 tons per sheep, on a wet basis. Therefore, the use of dairy cow fodder leftovers would potentially feed a production inventory from 146,832 to 183,540 sheep. In turn, this sheep inventory for meat production, with a conservative carcass yield of 45%, would generate a production value with annualized ranges of 14.4 to 18.1 million USD. This circular economy, animal productive efficiency, bioeconomic gains, and human well-being frame, would contribute to both the mitigation and resolutions of environmental and developmental issues.

## 5. Conclusions

This study, based on a rethink–reuse–reduce enquiry approach, bridged two noteworthy animal systems in northern Mexico, Comarca Lagunera: dairy cows and meat sheep schemes. Certainly, the use of feed-leftovers from the dairy cow industry as a basal diet aligned with the short-term supplementation with *Withania somnifera* promoted an enhanced testicular function, an enhanced seminal volume, while an amplified odor intensity and sexual behavior, either appetitive or consummatory, aligned with the improvement of some hemogram response variables across time in Black Belly rams. Such research outcomes should enhance not only the resilience and sustainability, but also speed up the sexual behavior and reproductive efficiency, of sheep production and should also heighten the well-being of sheep-producers and their families. Moreover, the research outcomes elicited by the short-term WS supplementation displayed an enhancement of ram´s reproductive and sexual function that may hold interesting implications from a translational standpoint.

## Figures and Tables

**Table 1:** Chemical and physical composition of the dairy cow feed-leftovers used as basal diet offered during the experimental period (40 d) to Black Belly rams (n = 20) in northern Mexico ^1,2,3,4^.

Ingredient	g kg^−1^ DM^−1^
Corn silage	241.6
Rolled corn grain	310.7
Alfalfa hay	135.2
Soybean meal	123.5
Cottonseed meal	94.5
Vitamins and minerals premix	94.5
Nutrient content of diet	%
Dry matter	80.0
Ash	7.3
Ether extract	3.8
Crude protein	15.3
Neutral detergent fiber	26.9
Acid detergent fiber	17.8
Starch	30.9
Total detergent nutrients	88.9
Metabolizable energy (Mcal kg^−1^ MS^−1^)	3.5

^1^ The short-term supplementation period included 40 days. ^2^ The presence of *Aspergillus flavus* in corn by-products was discarded [22]. ^3^ The metabolizable energy was calculated according to the equation previously proposed [23]. ^4^ The basal diet was collected daily (i.e., 0700 AM); every 10 d, 10 samples (i.e., 50 g each) were collected along the dairy cow feeding line and were subsequently chemically and physically analyzed.

**Table 2:** Least-square means ± standard error of mean for different corporal, testicular, and seminal response variables according to a short-term (40 d) supplementation with *Withania somnifera* either at low (LWS; 100 mg kg LW^−1^ d^−1^) or high (HWS; 200 mg kg LW^−1^ d^−1^) levels, or not supplemented (CONT), in Black Belly rams (n = 20) in northern Mexico (25° N).

Variable	CONT	LWS	HWS	*p*-Value
LW (kg)	55.3 ± 3.3 ^a^	56.8 ± 3.1 ^a^	54.1 ± 3.1 ^a^	0.84
BCS (u)	3.0 ± 0.05 ^a^	2.9 ± 0.07 ^a^	3.1 ± 0.05 ^a^	0.89
SCRC (cm)	32.4 ± 1.2 ^a^	32.9 ± 1.1 ^a^	28.25 ± 1.1 ^b^	0.01
ODOR (u)	0.0 ± 0.0 ^c^	0.2 ± 0.0 ^b^	0.4 ± 0.0 ^a^	0.01
LATEJA (s)	149.7 ± 47.5 ^a^	84.8 ± 44.0 ^a^	169.1 ± 44.0 ^a^	0.28
VOLEJA (mL)	0.2 ± 0.1 ^b^	0.7 ± 0.1 ^a^	0.3 ± 0.1 ^ab^	0.01
SPERCON (×10^6^ mL^−1^)	2480.1 ± 745.9 ^a^	2995.3 ± 690.6 ^a^	2272.4 ± 690.6 ^a^	0.72
MASMOT (units)	1.5 ± 0.7 ^a^	2.0 ± 0.7 ^a^	2.3 ± 0.7 ^a^	0.85
MASPROG (%)	31.6 ± 15.0 ^a^	39.6 ± 13.9 ^a^	43.9 ±13.9 ^a^	0.91
VIABILI (%)	30.4 ± 13.8 ^a^	33.9 ± 12.8 ^a^	35.7 ± 12.7 ^a^	0.98

Live weight (LW, kg), body condition score (BCS, units), scrotal circumference (SCRC, cm), odor (units), latency to ejaculation (LATEJA, s), volume ejaculated (VOLEJA, mL), sperm concentration (SPERCON, ×10^6^ mL^−1^), mass motility (MASMOT, units), mass progressive motility (MASPROG, %), sperm viability (VIABILI, %). ^a–c^ Least-square means without a common superscript within a response variable differ (*p* < 0.05).

**Table 3:** Least-square means ± standard error of mean for appetitive sexual behaviors, consummatory sexual behaviors, and other non-sexual behaviors according to a short-term (40 d) supplementation with *Withania somnifera* either at low (LWS; 100 mg kg LW^−1^ d^−1^) or high (HWS; 200 mg kg LW^−1^ d^−1^) levels, or not supplemented (CONT), in Black Belly rams (n = 20) in northern Mexico (25° N).

Variable	CONT	LWS	HWS	*p*-Value
***Appetitive sexual behavior***				
APPRO (n)	4.4 ± 2.4 ^a^	7.0 ± 2.2 ^a^	9.5 ± 2.2 ^a^	0.62
KICK (n)	2.6 ± 1.5 ^a^	5.4 ± 1.4 ^a^	3.0 ± 1.4 ^a^	0.48
ANGESNI (n)	5.3 ± 2.9 ^b^	4.7 ± 2.6 ^b^	14.3 ± 2.7 ^a^	0.03
CORPSNI (n)	2.9 ± 2.4 ^a^	4.5 ± 2.2 ^a^	8.8 ± 2.2 ^a^	0.22
FLE (n)	0.3 ± 0.5 ^a^	0.7 ± 0.5 ^a^	0.7 ± 0.5 ^a^	1.00
FLESNI (n)	1.6 ± 0.8 ^a^	1.5 ± 0.8 ^a^	1.3 ± 0.8 ^a^	0.99
FLEURSNI (n)	0.8 ± 0.5 ^a^	0.7 ± 0.5 ^a^	0.8 ± 0.5 ^a^	0.77
PENEXTR (n)	1.0 ± 1.4 ^a^	3.2 ± 1.3 ^a^	3.5 ± 1.3 ^a^	0.64
ATTMO (n)	0.8 ± 1.3 ^a^	2.6 ± 1.2 ^a^	3.3 ± 1.2 ^a^	0.61
***Consumattory sexual behavior***				
MOPENEXTR (n)	0.6 ± 1.3 ^bc^	1.8 ± 1.1 ^b^	4.2 ± 1.2 ^a^	0.04
MOPENINTRO (n)	1.6 ± 0.5 ^a^	1.5 ± 0.5 ^a^	1.4 ± 0.5 ^a^	0.56
MOEJACU (n)	0.9 ± 0.3 ^a^	0.5 ± 0.2 ^a^	0.9 ± 0.2 ^a^	0.69
***Other non-sexual behaviors***				
LVOCA (n)	0.7 ± 0.6 ^a^	0.8 ± 0.5 ^a^	2.1 ± 0.5 ^a^	0.25
HVOCA (n)	0.0 ± 0.4 ^a^	0.6 ± 0.4 ^a^	0.6 ± 0.4 ^a^	0.28
STAND (n)	0.3 ± 0.4 ^a^	0.8 ± 0.4 ^a^	0.1 ± 0.4 ^a^	0.58
ISOATTESC (n)	0.1 ± 0.1 ^a^	0.0 ± 0.1 ^a^	0.3 ± 0.1 ^a^	0.32

Appetitive sexual behavior [approaches (APRO, n), kicking (KICK, n), ano-genital sniffing (ANGESNI, n), corporal sniffing (CORPSNI, n), flehmen (FLE, n), flehmen + sniffing (FLESNI, n), fleshmen + urine sniffing (FLEURSNI, n), pennis extrusion (PENEXTR, n), attempt mounting (ATTMO, n)], consummatory sexual behavior [mount + pennis extrusion (MOPENEXTR, n), mount + pennis intromission (MOPENINTRO, n), mount + ejaculation (MOEJACU, n)], and other non-sexual behaviors [low vocalization (LVOCA, n), high vocalization (HVOCA, n), standing position (STAND, n), isolation + attempt to scape (ISOATTESC, n)]. ^a–c^ Least-square means without a common superscript within a response variable differ (*p* < 0.05).

**Table 4:** Least-square means ± standard error of mean for some hemogram response variables as affected by a short-term (40 d) supplementation with *Withania somnifera* either at low (LWS; 100 mg kg LW^−1^ d^−1^) or high (HWS; 200 mg kg LW^−1^ d^−1^) levels, or not supplemented (CONT), in Black Belly rams (n = 20) in northern Mexico (25° N).

Variable	CONT	LWS	HWS	*p*-Value
WBCC, (×10^9^ cells L^−1^)	113.7 ± 4.3 ^a^	121.8 ± 4.0 ^a^	121.9 ± 4.0 ^a^	0.32
RBCC, (×10^12^ cells L^−1^)	2.9 ± 0.1 ^a^	3.3 ± 0.1 ^a^	3.0 ± 0.1 ^a^	0.18
HbC, (g dL^−1^)	12.4 ± 0.4 ^b^	14.8 ± 0.4 ^a^	14.6 ± 0.4 ^a^	0.01
Ht, (%)	11.5 ± 0.7 ^a^	13.2 ± 0.7 ^a^	12.1 ± 0.7 ^a^	0.18
MCV, (fL)	39.5 ± 0.1 ^a^	39.9 ± 0.1 ^a^	39.9 ± 0.1 ^a^	0.17
MCHb, (pg)	48.7 ± 2.8 ^a^	45.9 ± 2.6 ^a^	49.4 ± 2.6 ^a^	0.65
MCHbC, (g dL^−1^)	124.8 ± 7.2 ^a^	115.5 ± 6.7 ^a^	124.4 ± 6.7 ^a^	0.61
RBCDW, (%)	14.5 ± 0.2 ^a^	14.2 ± 0.2 ^a^	14.5 ± 0.2 ^a^	0.44

White blood cell count (WBCC, ×10^9^ cells L^−1^), red blood cell count (RBCC, ×10^12^ cells L^−1^), hemoglobin concentration (HbC, g dL^−1^), hematocrit (Ht, %), mean corpuscular volume (MCV, fL), mean corpuscular hemoglobin (MCHb, pg), mean corpuscular hemoglobin concentration (MCHbC, g dL^−1^), and red blood cell distribution width (RBCDW, %). ^a,b^ Least-square means without a common superscript within a response variable differ (*p* < 0.05).

**Table 5:** Least-square means ± standard error of mean according to the supplementation-by-time interaction regarding the body condition score, scrotal circumference, odor, and volume ejaculated as affected by either a short-term (40 d) supplementation with *Withania somnifera* at low (LWS; 100 mg kg LW^−1^ d^−1^) or high (HWS; 200 mg kg LW^−1^ d^−1^) levels, or not supplemented (CONT), as well as the time supplementation period [i.e., d0 (Initial) or d40 (Final)] in Black Belly rams (n = 20) in northern Mexico (25° N) ^1^.

Variable	CONT		LWS		HWS		SEM ^2^	*p*-Value
	Initial	Final	Initial	Final	Initial	Final		
BCS (u)	3.0 ^b^	2.9 ^b^	2.9 ^b^	2.9 ^b^	3.0 ^b^	3.3 ^a^	0.1	0.01
SCRC (cm)	31.6 ^ab^	33.2 ^a^	31.7 ^ab^	34.0 ^a^	27.4 ^c^	29.0 ^bc^	1.2	0.01
ODOR (u)	0.0 ^c^	0.0 ^c^	0.0 ^c^	0.5 ^b^	0.0 ^c^	0.9 ^a^	0.0	0.01
VOLEJA (mL)	0.2 ^b^	0.3 ^b^	0.6 ^a^	0.8 ^a^	0.5 ^ab^	0.6 ^a^	0.1	0.01

^1^ Body condition score (BCS, units), scrotal circumference (SCRC, cm), odor (units), and volume ejaculated (VOLEJA, mL). ^2^ The most conservative standard error of the mean is presented. ^a–c^ Least-square means without a common superscript within a response variable differ (*p* < 0.05).

**Table 6:** Least-square means ± standard error of mean according to the supplementation-by-time interaction regarding appetitive sexual behaviors, consummatory sexual behaviors, and other non-sexual behaviors, as affected by either a short-term (40 d) supplementation with *Withania somnifera* either at low (LWS; 100 mg kg LW^−1^ d^−1^) or high (HWS; 200 mg kg LW^−1^ d^−1^) levels, or not supplemented (CONT), as well as the time supplementation period [i.e., d0 (Initial) or d40 (Final)] in Black Belly rams (n = 20) in northern Mexico (25° N) ^1^.

Variable	CONT		LWS		HWS		SEM ^2^	*p*-Value
	Initial	Final	Initial	Final	Initial	Final		
***Appetitive sexual behavior***								
APPRO (n)	1.8 ^b^	3.5 ^ab^	6.2 ^a^	4.5 ^ab^	4.0 ^ab^	2.1 ^ab^	1.7	0.01
KICK (n)	3.0 ^b^	5.8 ^ab^	6.5 ^ab^	7.5 ^ab^	7.4 ^ab^	11.5 ^a^	2.9	0.01
ANGESNI (n)	5.0 ^b^	5.6 ^b^	6.0 ^b^	3.4 ^b^	8.7 ^ab^	19.8 ^a^	3.5	0.01
CORPSNI (n)	1.8 ^b^	4.0 ^ab^	5.0 ^ab^	4.0 ^ab^	4.8 ^ab^	12.8 ^a^	3.2	0.01
FLE (n)	0.5 ^a^	0.1 ^a^	0.0 ^a^	1.4 ^a^	0.0 ^a^	1.4 ^a^	0.8	0.07
***Consumattory sexual behavior***								
MOPENEXTR (n)	0.1 ^b^	1.1 ^ab^	1.4 ^ab^	2.1 ^ab^	2.5 ^ab^	5.8 ^a^	1.5	0.01
MOPENINTRO (n)	0.6 ^ab^	1.6 ^a^	1.1 ^ab^	2.0 ^ab^	2.0 ^ab^	2.0 ^b^	0.07	0.03
MOEJACU (n)	0.3 ^b^	1.5 ^a^	0.4 ^b^	0.5 ^ab^	0.7 ^ab^	1.4 ^a^	0.03	0.04
***Other non-sexual behaviors***								
LVOCA (n)	1.3 ^b^	0.1 ^b^	0.5 ^b^	1.0 ^b^	0.8 ^b^	3.4 ^a^	0.7	0.01
ISOATTESC (n)	0.1 ^b^	0.0 ^b^	0.0 ^b^	0.0 ^b^	0.0 ^b^	0.7 ^a^	0.2	0.05

^1^ Appetitive sexual behavior [approaches (APRO, n), kicking (KICK, n), ano-genital sniffing (ANGESNI, n), corporal sniffing (CORPSNI, n), and flehmen (FLE, n), consummatory sexual behavior [mount + pennis extrusion (MOPENEXTR, n), mount + pennis intromission (MOPENINTRO, n), mount + ejaculation (MOEJACU, n)], and other non-sexual Behaviors [low vocalization (LVOCA, n), and isolation + attempt to scape (ISOATTESC, n)]. ^2^ The most conservative standard error of the mean is presented. ^a,b^ east-square means without a common superscript within a response variable differ (*p* < 0.05).

**Table 7:** Least-square means ± standard error of mean according to the supplementation-by-time interaction regarding the hemogram response variables as affected by either a short-term (40 d) supplementation with *Withania somnifera* either at low (SLW; 100 mg kg LW^−1^ d^−1^) or high (SHW; 200 mg kg LW^−1^ d^−1^) levels, or not supplemented (CONT), as well as the time supplementation period [i.e., d0 (Initial), d20 (Middle) or d40 (Final), in Black Belly rams (n = 20) in northern Mexico (25° N) ^1^.

Variables	CONT			LWS			HWS			SEM ^2^	*p*-Value
	Initial	Middle	Final	Initial	Middle	Final	Initial	Middle	Final		
WBCC (×10^9^ cells L^−1^)	113.6 ^b^	112.8 ^b^	114.8 ^b^	119.3 ^ab^	109.5 ^b^	136.6 ^a^	121.8 ^ab^	109.4 ^b^	134.6 ^a^	7.0	0.01
HbC (g dL^−1^)	11.7 ^b^	13.5 ^b^	12.2 ^b^	13.6 ^b^	13.6 ^a^	17.3 ^a^	13.0 ^b^	13.1 ^b^	17.7 ^a^	0.8	0.01
MCV (fL)	39.3 ^b^	39.2 ^b^	39.3 ^b^	39.3 ^b^	39.7 ^ab^	40.6 ^a^	39.4 ^b^	39.9 ^ab^	40.5 ^a^	0.3	0.01

^1^ White blood cell count (WBCC, ×10^9^ cells L^−1^), hemoglobin concentration (HbC, g dL^−1^), and mean corpuscular volume (MCV, fL). ^2^ The most conservative standard error of the mean is presented. ^a,b^ Least-square means without a common superscript within a response variable differ (*p* < 0.05).

## Data Availability

None of the data were deposited in an official repository, yet information can be made available upon request.
